# Effect of Dimethyl Sulfoxide Primer on Microtensile Bond Strength and Micromorphological Pattern of HEMA-free Universal Adhesive to Dry/Wet Dentin After Thermomechanical Aging 

**DOI:** 10.4317/jced.61252

**Published:** 2024-03-01

**Authors:** Asmaa A. Elbanna, Radwa I. El-Toukhy, Mohamed Abbas, Nadia M. Zaghloul

**Affiliations:** 1 Assistant Clinical Lecturer, Conservative Dentistry Department, Faculty of Dentistry, Mansoura University, Egypt; 2 Associate Professor, Conservative Dentistry Department, Faculty of Dentistry, Mansoura University, Egypt; 3 Associate Professor, Conservative Dentistry Department, Faculty of Dentistry, Horus University, New-Damietta, Egypt; 4 Professor, Dental Biomaterials Department, Faculty of Dental Medicine, Al-Azhar University for Boys, Cairo, Egypt; 5 Professor, Conservative Dentistry Department, Faculty of Dentistry, Mansoura University, Egypt

## Abstract

**Background:**

To evaluate the effect of dimethyl sulfoxide (DMSO) primer on microtensile bond strength (μTBS) and the micromorphological pattern of a hydroxyethyl methacrylate (HEMA)-free universal adhesive (UA) applied on wet/dry dentin in etch and rinse (E&R) mode before/after thermomechanical aging.

**Material and Methods:**

For the μTBS test, the mid-coronal dentin of 80 human mandibular first molars was exposed and etched with 35% phosphoric acid. Teeth were randomly divided into two equal groups: dry and wet dentin (n = 40). Then, each group was subdivided according to dentin pretreatment by DMSO before UA (Gluma Bond Universal, Heraeus Kulzer, Hanau, Germany) application into unpretreated and 10% DMSO/water (OT Primer S100, OT Oy Dent, Turku, Finland) pretreated (n = 20). Resin composite blocks were built up using a specially designed Teflon mold. In every subgroup, both the μTBS test and failure analysis by stereomicroscope were evaluated immediately after 24 h and after thermomechanical aging (n = 10). The data were statistically analyzed using a three-way analysis of variance (ANOVA) (*p* = 0.05). For the micromorphological pattern, 16 maxillary first premolars were distributed as mentioned in the μTBS test, prepared, and buccolingually sectioned. The dentin-resin interface was examined using an environmental scanning electron microscope (ESEM) (n = 2).

**Results:**

Three-way ANOVA revealed that the main effects and interactions between dentin wetness, dentin pretreatment, and evaluation time (thermomechanical aging) were not significant for µTBS (*p*> 0.05). Adhesive failure was the predominant type in all immediate and delayed specimens. Longer and more prominent resin tags were observed at dentin-resin interfaces after DMSO application.

**Conclusions:**

Neither the initial dentin wetness condition, dentin pretreatment, nor thermomechanical aging could affect the dentin bond strength. No correlation was found between the bond strength and the micromorphology findings.

** Key words:**Wet/dry dentin bonding, Microtensile bond strength, Micromorphology, Universal adhesive, Dimethyl sulfoxide, Thermomechanical aging.

## Introduction

The fast progress in dental adhesive technology over the last decades has extensively influenced modern restorative dentistry. Nevertheless, the bonded interface itself remains the Achilles heel of an adhesive filling, and the clinical longevity of resin composite restorations today is still too short. In this context, several aspects should be considered concerning the bond durability. These aspects include the heterogeneity of tooth structure composition and the features of the dental surface exposed following cavity preparation. The adhesive’s physicochemical features and how it interacts with the two substrates are two other factors to consider (1).

In adhesive dentistry, dentin bonding is regarded as a form of tissue engineering, which relies on hybrid layer formation. Unfortunately, the integrity of the hybrid layer faces challenges in the aging process. It has been shown that resin adhesion to dentin initially had a high bond strength after application but reduced by 50–60% after one to two years. Excessive demineralization by acid etching, hydrolysis of resin composites, insufficient adhesive monomer infiltration, and degradation of dentin collagen fibrils are the key reasons for hybrid layer degradation (2).

Universal adhesives are designed to be used in multiple modes (E&R, self-etch, or selective enamel etching), which makes them very versatile. In clinically relevant protocols, the E&R dentin bonding approach still uses the traditional wet-bonding technique to couple relatively hydrophilic adhesives to the hydrated dentin substrate. Despite this, adequate moisture management is not easily achieved. Either excess or lack of dentin moisture involvement within the hybrid layers may compromise resin-dentin bonding and make adhesive infiltration far from perfect (3). With excess moisture inclusion, water-associated artifacts may be manifested as water-infused hybrid/adhesive layers, bubbles, or discrete water films between the adhesive and resin composite. These water-associated artifacts not only increase interfacial permeability upon bonding but also promote phase separation of the adhesive components, polymer swelling, and leaching of resin components, degrading the mechanical properties. In the long run, water causes the hydrolysis of polymers containing ester linkages. This allows endogenous host-derived collagen hydrolytic enzymes (matrix metalloproteases (MMPs) and cysteine cathepsins (CTs)) to degrade demineralized collagen (4). As a result, the concept of dry bonding is thoroughly researched to reduce the amount of leftover water at the bonded interface without impairing the resin-dentin interaction. Nonetheless, concern remains regarding the inadequate resin-dentin interface caused by collagen collapse while air-drying the acid-etched dentin (5).

The mono-functional hydrophilic HEMA monomer is often added to UAs to enhance surface wetting and prevent phase separation. Nonetheless, HEMA encourages water sorption and hydrolysis. At adhesive interfaces, calcium nanolayering of 10-methacryloyloxydecyl dihydrogen phosphate (10-MDP) has been shown to be negatively inhibited by HEMA (6). However, without HEMA, the adhesive will be more susceptible to a phase-separation reaction between hydrophobic and hydrophilic components with water-tree nano-leakage formation within the polymerized adhesive layer (7). A study revealed that the type of solvent used in the adhesive formula is crucial for adjusting the surface wet pattern and improving the adhesive system’s performance (8). Acetone, in particular, evaporates much residual water. Though, since it cannot re-expand the shrunken collagen fibrils, it is more sensitive to air-drying. Acetone has a very high vapor pressure, which results in the joint effect on the residual water with the typical phase separation. The rapid evaporation may also not allow sufficient time for monomers to adequately infiltrate (9).

Recently, the application of DMSO ((CH3)2SO) to strengthen the dentin-resin bond has gained researchers’ interest. As a polar aprotic solvent, DMSO has a high ability to penetrate biological surfaces (4). With DMSO, the highly cross-linked collagen fibers can be separated into a sparser network, which facilitates resin diffusion (10). Moreover, DMSO can remove any remaining water from the dentin surface and thus, improves the wettability of demineralized dentin, reducing phase separation. Stape *et al*. (11) also reported that by disrupting the water layer bound to collagen molecules, DMSO promotes the exposure of more binding sites and allows more MDP molecules to bind with collagen. It also prevents collagen breakdown by blocking MMP-9 and MMP-2 enzymes (12). Different studies evaluated the effect of DMSO on immediate and long-term wet and dry dentin bond strength. They found that DMSO could improve and preserve the bond stability and hybrid layer integrity of different adhesive systems when used in high (10,11,13-15) or low (16-18) concentrations.

The findings of DMSO effects on UAs were few, inconsistent, and concentration-dependent. Therefore, the study aimed to evaluate and compare the effect of a 10% DMSO/water concentration on dentin μTBS and the micromorphology of a HEMA-free UA applied in E&R mode on wet and dry dentin. The null hypotheses tested were that 1) the initial dentin wetness condition would have no effect on the dentin μTBS, 2) irrespective of the initial dentin wetness condition, pretreatment with 10% DMSO/water would have no effect on bond strength to dentin, 3) thermomechanical aging would not influence the dentin μTBS, and 4) the micromorphology at the dentin-resin interface wouldn’t be affected by the different dentin wetness conditions, 10% DMSO/water application, and thermomechanical aging.

## Material and Methods

Materials used in the study, with their full description and details, are shown in Table 1.

**Table 1:**
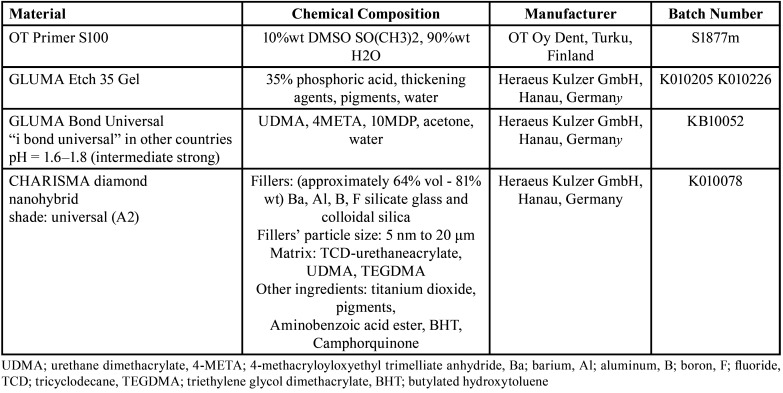
Materials used in the study.

-Teeth selection and preparation

Based on a protocol (No. A14020822) approved by an ethical committee at Mansoura University, 80 freshly extracted intact human permanent mandibular first molars and 16 maxillary first premolars were extracted from healthy patients (aged 45 to 55 years) due to periodontal causes. Teeth were cleaned using an ultrasonic scaler (Guilin Woodpecker Medical Instrument Co., Ltd., Guangxi, China), disinfected in 0.5% chloramine-T for 24 h, and stored in distilled water at 4 °C for no longer than three months until usage.

The selected teeth were centrally embedded in cylindrical containers of 29 mm internal diameter and 35 mm height with the aid of a centralizing device. These cylinders were filled with epoxy resin (Kemapoxy150 3D, CMB Co., Wadi El Natroun, Egypt) up to 2 mm below the CEJ. The occlusal part of the tooth was removed under water coolant until the mid-coronal dentin was exposed using a low-speed automated diamond saw (PICO 155 Precision Saw, PACE TECHNOLOGIES, Tucson, USA) in a direction perpendicular to the tooth’s longitudinal axis. The cut surface was ground using 600-grit silicon carbide paper (1913 Siawat, Sia Abrasives, Frauenfeld, Switzerland) in a circular motion for 30 s in the presence of water coolant to achieve a standardized smear layer with proper thickness.

-Microtensile bond strength test

-Sample size calculation and experimental design

Sample size calculation was achieved using Power and Sample Size Calculation Software (Version 3.1.2, Vanderbilt University, Tennessee, USA). Based on a previous paper by Cardenas *et al*. (9), the expected mean difference in dentin µTBS between the main two groups (with and without primer) was 8±2.7 MPa. Using a power of 99% and a 5% significance level, a total of at least six samples in each group was needed. This number was to be increased to eight samples in each group (25% more than calculated) to compensate for possible losses, damages, and failures during work. After that, to make sure of the data results, the sample size was increased to ten samples per group (80 total). As this study was based on three variables defined as (1) initial dentin wetness conditions at two levels (wet and dry dentin), (2) dentin pretreatment with DMSO at two levels (no pretreatment and 10% DMSO/water), and (3) evaluation time (thermomechanical aging) at two levels (immediate (no aging) and delayed (after aging), the eighty mandibular first molars were randomly divided into two main groups (n = 40) according to the initial dentin wetness condition. Then, each group was subdivided into two subgroups according to surface pretreatment (n = 20). Specimens of each subgroup were tested immediately or after aging (n = 10).

-Bonding procedure and resin composite block building up

**Figure 1:**
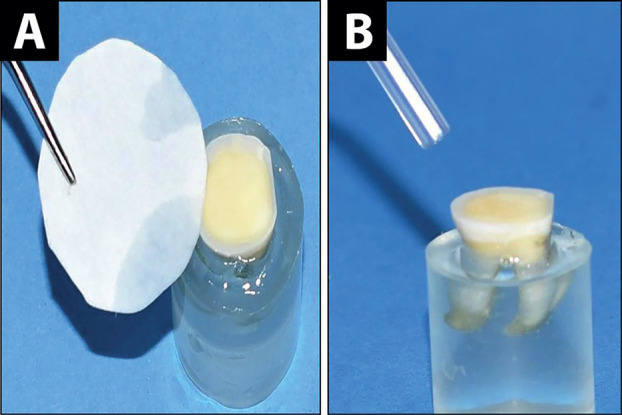
Dryness of the acid-etched dentin surface A Blot dryness, B Air dryness.

The dentin surface was etched with 35% phosphoric acid (Gluma etch 35 gel, Heraeus Kulzer, Hanau, Germany) for 15 s, then rinsed with water for 30 s. Dryness of the surface was done either by blot dryness using filter paper (Double Ring 102 Qualitative Filter Paper, Shenyang Great Wall Filtration CO., Shenyang, China), leaving a partially wet dentin surface, or by air for 30 s using an oil-free air flow three-way syringe held at a 45-degree angle at a distance of approximately 10 cm under maximum pressure (dry dentin), (Fig. 1).

Materials were applied according to the different manufacturers’ instructions, and the same LED light curing device (Elipar, 3M ESPE, St. Paul, Minnesota, USA) delivering 1200 mW/cm² was used throughout the experiment. The light cure device’s intensity was measured every five specimens by a radiometer (Bluephase Meter II, Ivoclar Vivadent, NY, USA). In the case of the unpretreated (control) groups, the UA (GLUMA Bond Universal, Heraeus Kulzer, Hanau, Germany) was applied with no dentin pretreatment. One drop of the adhesive was dispensed in a mixing well and used immediately within 3 minutes. One coat was applied and actively rubbed over the surface for 20 s using a microbond brush. The surface was carefully air-dried with gentle oil-free air flow until the adhesive film no longer moved by moving the airflow from outside to inside while increasing the intensity at the same time. A glossy surface was obtained after adhesive application and excess solvent evaporation. The adhesive layer was then light-cured for 10 s. In the DMSO-pretreated groups, one drop of 10% DMSO/water (OT Primer S100, OT Oy Dent, Turku, Finland) was dispensed in a mixing well like that for the adhesive. One coat was applied and actively rubbed on the dentin surface for 60 s. An air stream was gently applied to remove the excess primer for 30 s through an air syringe at a distance of 10 cm. After that, the UA was applied.

**Figure 2:**
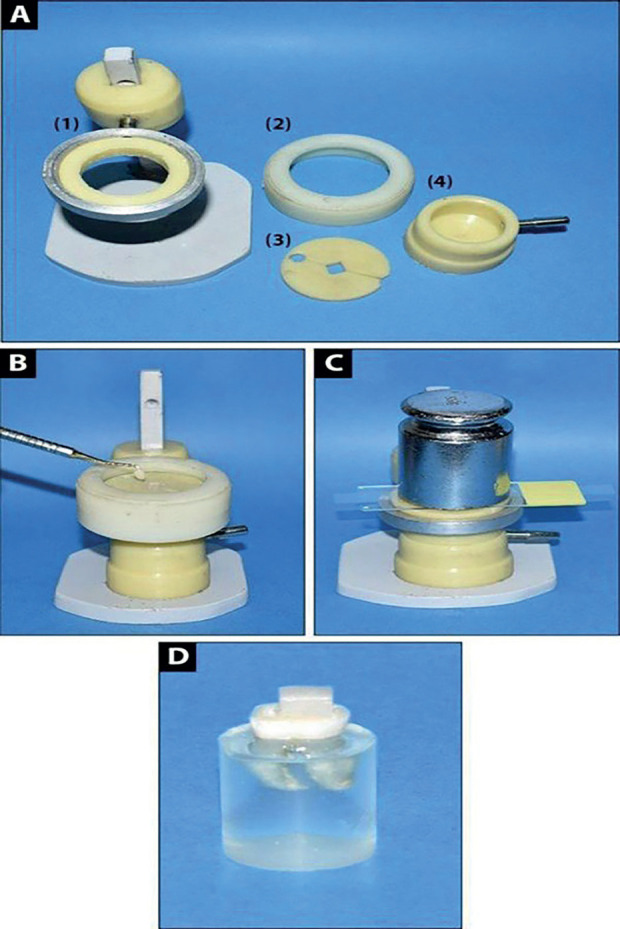
Resin composite application A Components of the customized device used in resin composite application; (1)- A metallic ring used as a fixture to hold and centralize the mold over the bonding surfaces as resin composite was being applied. It was hanged on a metallic pole to fit all blocks’ heights (2)- A Teflon governor was supplied to stabilize the spilt mold in its place during restoration insertion (3)- A rounded spilt Teflon mold with a central square hole (6 mm x 6 mm and 4 mm in height) (4)- A Teflon base constructed to involve the epoxy resin blocks for more support and prevention of specimen displacement upon resin composite application, B Resin composite application using a gold-platted composite applicator, C 500-g weight applied over the glass slide for better resin composite adaptation, D Restoration’s final view.

To standardize resin composite block building up, a specially designed rounded spilt Teflon mold with a central square hole (6 mm x 6 mm and 4 mm in height) was constructed. The Teflon mold was centralized over the bonding surfaces with the aid of a customized device (Fig. 2). Resin composite blocks (CHARISMA diamond, Heraeus Kulzer, Hanau, Germany) were built up in two equal increments to a total height of 4 mm using a gold-plated metallic composite applicator (Miltex GmbH, Viernheim, Germany). Each increment was packed well and light-cured for 20 s. To ensure a smooth superficial surface and better resin composite accommodation, a transparent polyester Mylar strip of 10 mm width (TOR-VM Ltd., Moscow, Russia) was adapted to the surface of the final increment. Then, a thin transparent glass microscope slide and a 500-g weight were placed on the strip and left for 30 s. After that, both the weight and glass slide were removed, and the surface was light-cured with the light tip in close contact with the polyester strip. Following resin composite application, specimens were kept in distilled water for 24 h in an incubator (BTC, Model: BT1020, Cairo, Egypt) at 37 °C to allow water sorption and complete the post-operative polymerization reaction. Then, half of the specimens from each subgroup were tested immediately, while the other half was subjected to thermomechanical aging before being tested.

-Thermomechanical aging

For thermomechanical aging, a programmable logic controlled equipment; the newly developed four-station multi-modal ROBOTA chewing simulator (ROBOTA Model ACH-09075DC-T, Ltd., AD-Tech Technology Co., Frankfurt, Germany) coupled with a thermo-cyclic protocol operated on a servo-motor was used. A weight of 5 kg, which is equivalent to 49 N of chewing force, was exerted with thermal aging in a cold/hot water bath, as illustrated in Table 2. The test was conducted 150000 times to clinically simulate the chewing condition for a year, according to a previous systematic review (19).

**Table 2:**
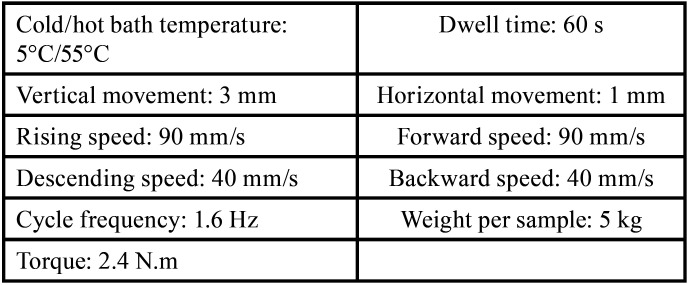
Thermomechanical aging guidelines.

-Microtensile bond strength testing procedures and failure mode analysis

The specimens were cut using a low-speed automated diamond disc (Isomet 4000, Buehler Ltd., Lake Bluff, IL, USA) under copious water coolant to obtain dentin-resin composite beams that were 1 x 1 mm in dimensions. A digital caliper (Mitutoyo, Tokyo, Japan) was used to check the beams’ dimensions. From each specimen, six defect-free central beams were selected to be tested, while peripheral beams were excluded. Beams were mounted onto a universal testing machine (Instron model 3345, MA, USA) using Gerald Eli’s jig. A tensile load was applied at a cross-head speed of 0.5 mm/min until bond failure occurred. For the analysis, each tooth served as the statistical unit. The bond strength value of each separate tooth was represented by the mean value of the beams obtained from that tooth and calculated in Mega Pascal (MPa) (Bluehill Lite 3 software, version 3.3, Instron, Norwood, MA, USA). Specimens that displayed premature failure were excluded from the statistical analysis.

A stereomicroscope (Nikon SMZ745T, Tokyo, Japan) at a magnification of 30x was used to determine the mode of failure. Failure modes were classified as cohesive (when more than 75% of the failure was in composite or in dentin), adhesive (when more than 75% of the failure was at the dentin-resin interface), or mixed (when a mixture of adhesive and cohesive failure patterns occurred in the same sample).

-Micromorphological pattern analysis of the adhesive interface (descriptive analysis)

The 16 prepared maxillary first premolars were assigned to 8 experimental groups (n = 2) and restored according to the experimental design and restorative procedure mentioned in the μTBS test. The universal (Tofflimire) matrix system (WaterPik Technologies, Inc., California, USA) was used for resin composite application instead of the Teflon mold. Teeth were first cut buccolingually into two equal halves. Next, each half was polished using silicone carbide paper (1913 Siawat, Sia Abrasives, Frauenfeld, Switzerland) in four different grit sizes: coarse (600 grit), medium (800 grit), fine (1000 grit), and ultrafine (1200 grit, 1500 grit, 2000 grit, and 2500 grit). Then, fine diamond pastes with particle sizes of 3 μm, 1 μm, and 0.5 μm, respectively (ENA HRI polishing system, Micerium S.p.A., Genova, Italy) and a polishing brush (ENA HRI polishing brushes, Micerium S.p.A., Genova, Italy) were used for final polishing. After that, specimens were cleaned for 10 min in an ultrasonic bath (CD-4820 CODYSON Digital Ultrasonic Cleaner, Shenzhen, China). Finally, specimens were exposed to a 10% orthophosphoric acid solution for 5 s to demineralize all dentin collagen fibers and then to a 5% sodium hypochlorite solution for 5 min to remove the organic components. The specimens were prepared for ESEM (Prisma E model, Thermo Fisher Scientific Inc., Waltham, Massachusetts, USA), fixed on aluminum stubs with a standard diameter using carbon double sticky tape, and imaged at 2000x magnification with an accelerating voltage of 30 kV. Representative images of different specimens were selected.

-Statistical Analysis

All the µTBS obtained data were collected, tabulated, and coded using Microsoft Excel 365 Spreadsheet Software (Microsoft Corporation, Redmond, WA, USA). Data analysis was done using Statistical Package for Social Science (SPSS Software, Version 25, IBM, Chicago, IL, USA). Shapiro–Wilk test of normality was performed to detect the normal distribution of the raw data. As the results of this test revealed that all the data were normally distributed, parametric statistics (three-way ANOVA) was done.

## Results

-Microtensile bond strength 

According to the three-way ANOVA test findings, none of the main effects or interaction terms between variables (dentin wetness, primer application, aging) and µTBS measurements were statistically significant (*p* > 0.05), indicating that all variables did not have significant effects on the µTBS. The means, standard deviations, minimum, median, maximum, and *p* values of the µTBS of dental bonds under different conditions are presented in Table 3.

**Table 3:**
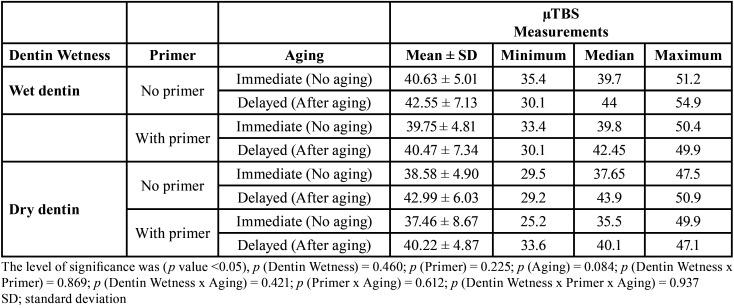
Means, standard deviations, minimum, median, maximum, and p values of the µTBS of all tested groups.

-Failure mode analysis 

Using the Chi-square test, the evaluation of failure modes was based on four modes expressed in frequency and percentage, as listed in  Table 4. In all the immediate and delayed groups, the failure mode patterns were predominantly adhesive.

**Table 4:**
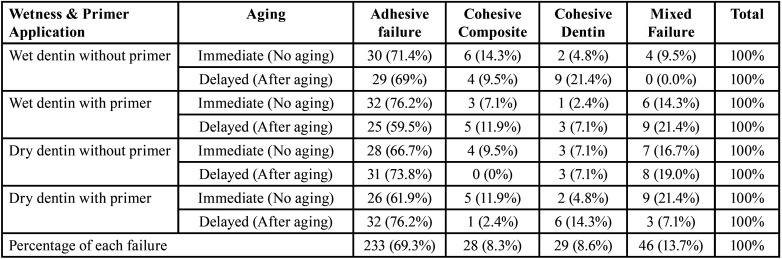
Frequency and percentage of failure modes in all tested groups.

-Micromorphological pattern analysis at the dentin-resin interface

**Figure 3:**
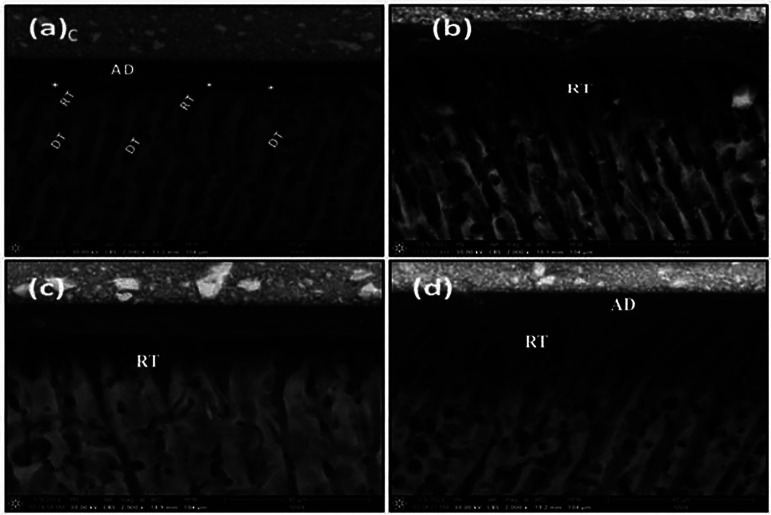
Representative ESEM images for the evaluation of the wet dentin groups (a) The un-pretreated group on immediate evaluation, (b) The DMSO-pretreated group on immediate evaluation, (c) The un-pretreated group on delayed evaluation, (d) The DMSO-pretreated group on delayed evaluation, C; composite, AD; adhesive layer, RT; resin tags, DT; dentinal tubules, White asterisk; gap.

The descriptive ESEM Figures of the dentin-resin interface showed the following:

• Wet dentin groups (Fig. 3): On immediate evaluation, the unpretreated group (Fig. 3a) showed uniform resin tags, while some samples showed microleakage (a gap) between the adhesive layer and dentin. The pretreated group (Fig. 3b) showed resin tags extended inside the dentinal tubules that were prominent, long, funnel-shaped, and compact.

On delayed evaluation after thermomechanical aging, the unpretreated group (Fig. 3c) showed resin tags that were short, thin, and oriented in different directions, while in the pretreated group (Fig. 3d), the resin tags were more prominent and contiguous with the adhesive layer, which was uniformly adherent to the dentin surface, and no gaps were seen.

**Figure 4:**
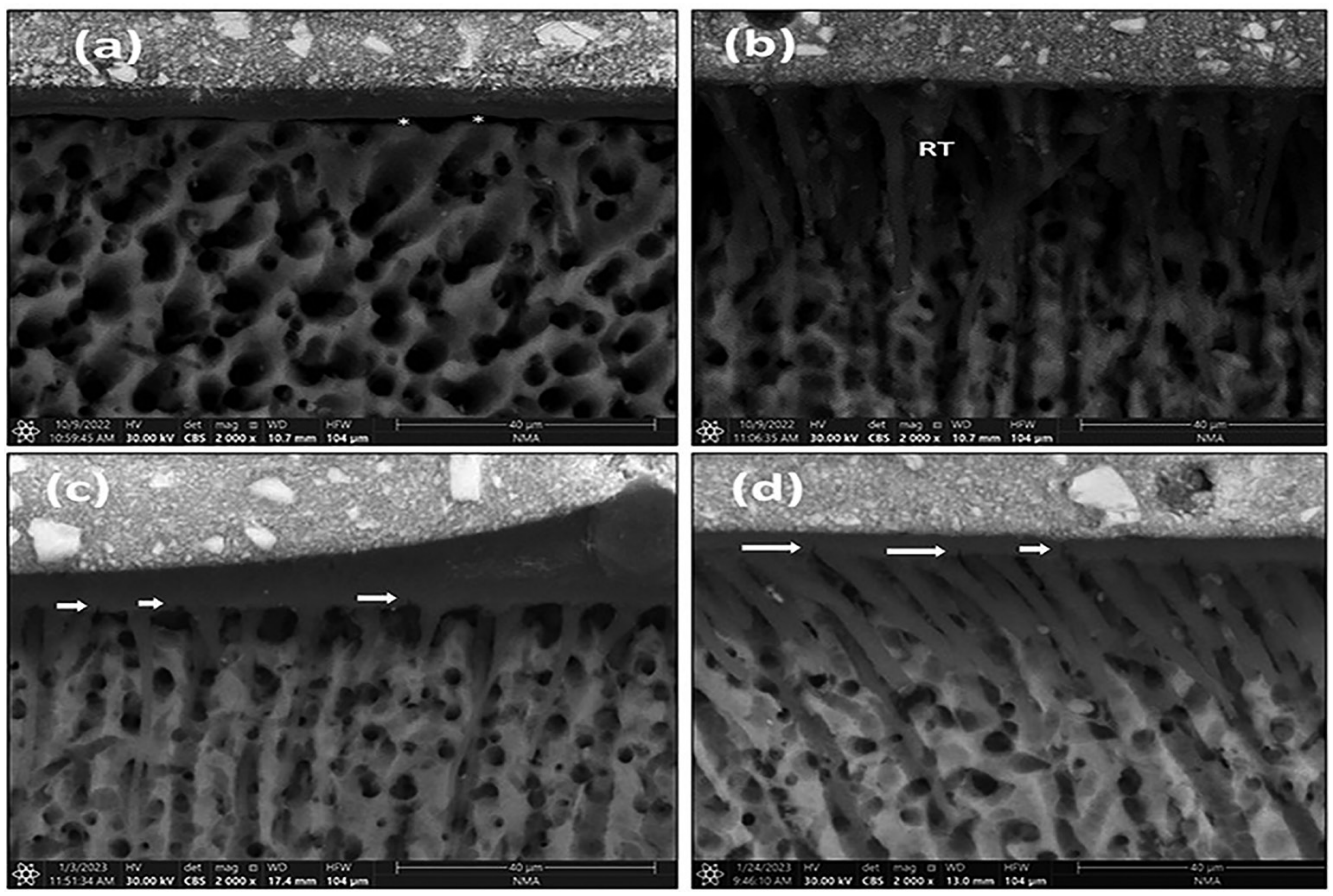
Representative ESEM images for the evaluation of the dry dentin groups (a) The unpretreated group on immediate evaluation, (b) The DMSO-pretreated group on immediate evaluation, (c) The unpretreated group on delayed evaluation, (d) The DMSO-pretreated group on delayed evaluation RT; resin tags, White asterisk; gap, White arrows; crack in hybrid &adhesive layers.

• Dry dentin groups (Fig. 4): When compared with their comparable wet groups, fewer resin tags and a thinner adhesive layer in some specimens were seen.

On immediate evaluation, the unpretreated group (Fig. 4a) showed thin, extremely short, fractured, or even absent resin tags. In the pretreated group (Fig. 4b), these resin tags were greater in number and length.

On delayed evaluation after thermomechanical aging, cracks (nano-cracks) in the adhesive layer were seen. In the unpretreated group (Fig. 4c), the resin tags were thin and short, with some gaps seen. In the pretreated group (Fig. 4d), the resin tags were prominent, long, and funnel-shaped, but no gaps were found.

## Discussion

The durability of the adhesive bond in restorative dentistry is crucial for the restoration’s longevity and clinical performance. Unfortunately, challenges persist in maintaining this durability, rendering it limited. The combination of resin component hydrolysis, phase separation, MMPs and CTs-induced collagenolytic activity, temperature, bacterial acids, microleakage, and mechanical stresses compromise the long-term interfacial integrity of the HL in bonded restorations (20). Hence, many new formulations and compositions of dental adhesives are being developed to increase bond strength and durability.

As a versatile and reliable testing method, the μTBS test was used to evaluate the durability of dentin-resin bonds under different conditions. As the bond strength of resin composites to dentin that is above or close to the pulp is around 30-40% of that to peripheral dentin, peripheral beams were excluded, and central beams were selected for more standardization and to decrease regional and tooth variabilities (21). According to the results of the present study, the null hypotheses regarding the bond strength were accepted since the main effects and interactions between the tested variables had no significant effect on dentin µTBS.

The first null hypothesis was accepted, as the dry dentin bonding protocol didn’t affect the bond strength compared to the wet dentin bonding protocol. This might be due to the long application time of Gluma Bond Universal (20s) together with the presence of water as a constituent of the adhesive, which is needed for acidic monomer ionization and was enough to re-expand the collagen fibrils. The active rubbing motion used for adhesive application was enough to increase the moieties kinetics and allow better monomer diffusion inwards (22). Such findings were in agreement with Sebold *et al*. (23) and Choi *et al*. (24), despite the acetone content of the used UAs. Reis *et al*. (22) also found that when acetone-based systems are agitated on the dentin surface, strong dentin bond strength can be produced in wet and dry dentin conditions. Another reason could be the presence of the 10-MDP functional monomer in UAs. The collagen fibers are preserved by the subsequent deposition of MDP-calcium salts, which have a lower solubility than salts generated from other functional monomers. The presence of 4-META as a functional monomer in GBU might also strengthen the binding between dentin and the adhesive formulation of 10-MDP. This is likely due to the bonding of its derived carboxylic acid to collagen uncovered by 10-MDP (25). This was in accordance with Marchesi *et al*. (26) who investigated the adhesive stability over time of a multi-mode, one-step adhesive applied using the E&R mode. They found a similar performance for the applied 10-MDP containing UA on both dry and wet dentin by aging for 24 h, six months, and one year in artificial saliva. Also, Leite *et al*. (27) found the same outcomes regarding the immediate bond strength of a UA applied in E&R mode to both dry and wet dentin and contributed what occurred to the 10-MDP content of the used UA. However, Dutra *et al*. (28) found a higher dentin μTBS of a 10-MDP containing UA when used with the conventional adhesive strategy on moist dentin than on dry dentin.

The use of DMSO didn’t affect the dentin bond strength of the UA. This might be because 10% DMSO was too weak to make any increase in the bond strength. Also, the reduced availability of crosslinking dimethacrylate monomers in UA, which only allowed their penetration with DMSO, limited the improvement of bond strength to the same extent as in simplified E&R adhesives, as shown by Stape *et al*. (4) Moreover, the water and (25–50%) acetone content in Gluma Bond Universal, together with the 10% DMSO/90% water and water left from E&R bonding, might have hindered the evaporation of solvents in UA. As a polar aprotic compound that can absorb water as it is characteristically attracted to hydrogen molecules, dentin pretreatment with DMSO/water might contribute to the residual water that not only hindered the evaporation of the DMSO solvent but also made it unable to improve dentin µTBS (accepted second null hypothesis) (29). Excess solvents and water at the resin-dentin interface can dilute the monomer and inhibit the polymerization reaction.

Owing to its potential for imitating mastication, thermomechanical aging was recommended for restoration aging. Epoxy resin was chosen for tooth mounting because of its good mechanical properties that would resist mechanical fracture on force application. Regarding the third null hypothesis, it was also accepted that thermomechanical aging couldn’t negatively affect the dentin µTBS. This might be due to the chemical bonding ability of the 10-MDP and 4-META content of the UA, which might also be responsible for resisting thermomechanical aging, making no significant difference in bond strength value between immediate and delayed (aged) specimens. However, this finding was inconsistent with a previous study (30) that tested the dentin bond strength of four different UAs in E&R mode before and after thermocycling. The study showed that no difference was detected before thermocycling between the tested adhesives. Thermocycling only affected a HEMA-free 10-MDP/acetone-containing UA. One explanation for what occurred might be due to the moisture control, the application mode used in the study, or the oxygen-inhibiting layer.

Regarding the failure mode, adhesive failure was the most frequently observed failure mode, indicating an inadequate bond at the dentin interface. It was difficult to compare the results of the present study with those of the previous ones as different adhesive formulas, DMSO concentrations, aging procedures, and exposure times were used. No previous study used 10% DMSO on UAs. Only 50% DMSO (4,29) was found to have no immediate or delayed effect on the bond strength, while 50% and 1% DMSO (9,31) were able to increase the immediate and delayed bond strength in other studies. On the other hand, 50% DMSO was found to significantly decrease the bond strength of a UA used by Mirzaei *et al*. (12).

Choi *et al*. (24) confirmed how different dentin surface moisture affected the performance of adhesives used in E&R mode: the resin tags were lower and with an irregular pattern, the hybrid layer thickness was not uniform, and the penetration depth was lower. In the study, the dry dentin group showed fewer and thinner resin tags with decreased penetration depth when compared to the wet dentin group, which was consistent with Tsujimoto *et al*. (3), although a bond with dentin was gained. This could indicate that there is no relation between the length of resin tags or hybrid layer thickness and the final bond strength, as illustrated by Rahal *et al*. (32).

An increase in length, number, and penetration depth of resin tags into dentin was seen on both wet and dry dentin after DMSO application. These findings might be related to the wetting and penetration-enhancing effects of DMSO as well as its ability to re-expand the collapsed dentin collagen. Such findings agreed with Guo *et al*. (14). The effect of thermomechanical aging on specimens was seen by the formation of cracks at the adhesive/hybrid layer, which correlates to the increase in adhesive failure, especially in the dry dentin groups.

Although this in-vitro study is a reliable method of comparison, it can’t completely simulate the oral environment as other factors (ex., saliva, changing pH) can affect the study outcomes. For this reason, in-vivo studies should be conducted to help assess the clinical success and sustainability of tested materials. Another important limitation of the current study is that the effect of 10% DMSO on HEMA-free UAs with acetone content was not compared with its effect on HEMA/ethanol-containing ones, HEMA-free simplified E&R adhesives, or non-simplified adhesive systems. For this reason, further future investigations on the effect of 10% DMSO on other adhesive formulas with different storage media and times are still required.

## References

[1] Van Meerbeek B, Peumans M, Poitevin A, Mine A, Van Ende A, Neves A (2010). Relationship between bond-strength tests and clinical outcomes. Dent Mater.

[2] Breschi L, Maravic T, Cunha SR, Comba A, Cadenaro M, Tjäderhane L (2018). Dentin bonding systems: From dentin collagen structure to bond preservation and clinical applications. Dent Mater.

[3] Tsujimoto A, Shimatani Y, Nojiri K, Barkmeier WW, Markham MD, Takamizawa T (2019). Influence of surface wetness on bonding effectiveness of universal adhesives in etch-and-rinse mode. Eur J Oral Sci.

[4] Stape THS, Tjäderhane L, Abuna G, Sinhoreti MAC, Martins LRM, Tezvergil-Mutluay A (2018). Optimization of the etch-and-rinse technique: New perspectives to improve resin-dentin bonding and hybrid layer integrity by reducing residual water using dimethyl sulfoxide pretreatments. Dent Mater.

[5] Pashley DH, Tay FR, Carvalho RM, Rueggeberg FA, Agee KA, Carrilho M (2007). From dry bonding to water-wet bonding to ethanol-wet bonding. A review of the interactions between dentin matrix and solvated resins using a macromodel of the hybrid layer. Am J Dent.

[6] Wendlinger M, Nuñez A, Moreira P, Carneiro TS, Cochinski GD, Siqueira F (2023). Effect of the Absence of HEMA on the Bonding Properties of Universal Adhesive Systems Containing 10-MDP: An In Vitro Study. Oper Dent.

[7] Mahdan MH, Nakajima M, Foxton RM, Tagami J (2013). Combined effect of smear layer characteristics and hydrostatic pulpal pressure on dentine bond strength of HEMA-free and HEMA-containing adhesives. J Dent.

[8] de Solventes CA (2009). Adhesive systems: Considerations about solvents. Int J Odontostomatol.

[9] Cardenas AFM, Araujo LCR, Szesz AL, de Jesus Tavarez RR, Siqueira F, Reis A (2021). Influence of application of dimethyl sulfoxide on the bonding properties to eroded dentin. J Adhes Dent.

[10] Stape THS, Tjäderhane L, Marques MR, Aguiar FHB, Martins LRM (2015). Effect of dimethyl sulfoxide wet-bonding technique on hybrid layer quality and dentin bond strength. Dent Mater.

[11] Stape THS, Tjäderhane L, Tezvergil-Mutluay A, Yanikian CRF, Szesz AL, Loguercio AD (2016). Dentin bond optimization using the dimethyl sulfoxide-wet bonding strategy: A 2-year in vitro study. Dent Mater.

[12] Mirzaei K, Ahmadi E, Rafeie N, Abbasi M (2023). The effect of dentin surface pretreatment using dimethyl sulfoxide on the bond strength of a universal bonding agent to dentin. BMC Oral Health.

[13] Stape THS, Mutluay MM, Tjäderhane L, Uurasjärvi E, Koistinen A, Tezvergil-Mutluay A (2021). The pursuit of resin-dentin bond durability: Simultaneous enhancement of collagen structure and polymer network formation in hybrid layers. Dent Mater.

[14] Guo J, Lei W, Yang H, Zhang Y, Zhao S, Huang C (2017). Dimethyl sulfoxide wet-bonding technique may improve the quality of dentin bonding. J Adhes Dent.

[15] Stape THS, Uctasli M, Cibelik HS, Tjäderhane L, Tezvergil-Mutluay A (2021). Dry bonding to dentin: Broadening the moisture spectrum and increasing wettability of etch-and-rinse adhesives. Dent Mater.

[16] Tjäderhane L, Mehtälä P, Scaffa P, Vidal C, Pääkkönen V, Breschi L (2013). The effect of dimethyl sulfoxide (DMSO) on dentin bonding and nanoleakage of etch-and-rinse adhesives. Dent Mater.

[17] Al-Ani AAS, Mutluay M, Stape THS, Tjäderhane L, Tezvergil-Mutluay A (2018). Effect of various dimethyl sulfoxide concentrations on the durability of dentin bonding and hybrid layer quality. Dent Mater J.

[18] Saffarpour A, Valizadeh S, Amini A, Kharazifard MJ, Rohaninasab M (2020). Effect of matrix metalloproteinase inhibitors on microtensile bond strength of dental composite restorations to dentin in use of an etch-and-rinse adhesive system. Clin Exp Dent Res.

[19] Nawafleh N, Hatamleh M, Elshiyab S, Mack F (2016). Lithium Disilicate Restorations Fatigue Testing Parameters: A Systematic Review. J Prosthodont.

[20] Braga RR, Fronza BM (2020). The use of bioactive particles and biomimetic analogues for increasing the longevity of resin-dentin interfaces: A literature review. Dent Mater J.

[21] Harp YS, Montaser MA, Zaghloul NM (2022). Flowable fiber-reinforced versus flowable bulk-fill resin composites: Degree of conversion and microtensile bond strength to dentin in high C-factor cavities. J Esthet Restor Dent.

[22] Reis A, Pellizzaro A, Dal-Bianco K, Gones OM, Patzlaff R, Loguercio AD (2007). Impact of adhesive application to wet and dry dentin on long-term resin-dentin bond strengths. Oper Dent.

[23] Sebold M, Giannini M, André CB, Sahadi BO, Maravic T, Josic U (2022). Bonding interface and dentin enzymatic activity of two universal adhesives applied following different etching approaches. Dent Mater.

[24] Choi AN, Lee JH, Son SA, Jung KH, Kwon YH, Park JK (2017). Effect of Dentin Wetness on the Bond Strength of Universal Adhesives. Materials (Basel).

[25] Iwai H, Fujita K, Iwai H, Ikemi T, Goto H, Aida M (2013). Development of MDP-based one-step self-etch adhesive--effect of additional 4-META on bonding performance. Dent Mater J.

[26] Marchesi G, Frassetto A, Mazzoni A, Apolonio F, Diolosà M, Cadenaro M (2014). Adhesive performance of a multi-mode adhesive system: 1-year in vitro study. J Dent.

[27] Leite M, Costa CAS, Duarte RM, Andrade AKM, Soares DG (2018). Bond Strength and Cytotoxicity of a Universal Adhesive According to the Hybridization Strategies to Dentin. Braz Dent J.

[28] Dutra DJ, Branco NT, Alvim HH, Magalhães CS, Oliveira RR, Moreira AN (2022). Bond strength of two universal adhesive systems to human dentin using different strategies. Acta Odontol Latinoam.

[29] Mello RMM, Alcântara BAR, França FMG, Amaral F, Basting RT (2022). Dimethyl Sulfoxide Dentin Pretreatments Do Not Improve Bonding of a Universal Adhesive in Etch-and-Rinse or Self-etch Modes. J Adhes Dent.

[30] Jacker-Guhr S, Sander J, Luehrs A K (2019). How "universal" is adhesion? Shear bond strength of multi-mode adhesives to enamel and dentin. J Adhes Dent.

[31] Zabeu GS, Giacomini MC, Scaffa PMC, Tjäderhane L, Mosquim V, Wang L (2023). Solvation role of dimethyl sulfoxide on the interaction with dentin bonding systems after 30 months. Dent Mater.

[32] Rahal V, Briso AL, dos Santos PH, Sundefeld ML, Sundfeld RH (2011). Influence of the hybrid layer thickness and resin tag length on microtensile bond strength. Acta Odontol Latinoam.

